# Relevance and Timing of Implant‐Driven Rehabilitation in Central Giant Cell Granuloma Cases—A Scoping Review

**DOI:** 10.1002/cre2.70085

**Published:** 2025-02-12

**Authors:** Roman Tatiana, Robert Thomas, Leclercq Olivier, Nafash Gilbert, Kharouf Naji, Olivier Etienne, Boschin Francois

**Affiliations:** ^1^ Department of Prosthodontics, Dental Faculty University of Strasbourg Strasbourg France; ^2^ INSERM UMR_S 1121, CNRS EMR 7003, Biomaterials and Bioengeneering University of Strasbourg Strasbourg France; ^3^ Pôle de Médecine et Chirurgie Bucco‐Dentaire, Hôpital Civil, Hôpitaux Universitaire de Strasbourg Strasbourg France; ^4^ Private practice Labège France; ^5^ Former Intern Dental Faculty University of Lille Lille France; ^6^ Private practice Arras France; ^7^ Formerly Department of Periodontology and Implantology, Dental Faculty University of Lille France; ^8^ Service d'Odontologie Abel Caumartin, Pôles Spécialités Médico‐chirurgicales, Centre Hospitalier Universitaire de Lille France; ^9^ Department of Operative Dentistry and Endodontics, Dental Faculty University of Strasbourg Strasbourg France; ^10^ Private practice Strasbourg France; ^11^ Private practice Lille France; ^12^ Department of Periodontology and Implantology, Dental Faculty University of Lille France

**Keywords:** central giant cell granuloma, implant, implant supported protheses, scoping review

## Abstract

**Objectives:**

Central giant cell granuloma (CGCG) is a rare benign tumor. Extended aggressive lesions require large resections, which can lead to bone defects and tooth loss. Rehabilitative treatment is necessary to restore good aesthetics and function. However, the protocol for implant treatment post‐CGCG is still unclear. The objective of this scoping review is to shed light on the rehabilitation protocol for CGCG sites by outlining the relevance and timing of implant surgery and prosthetic rehabilitation.

**Materials and methods:**

The review followed the Preferred Reporting Items for Systematic Review and Meta‐Analyses statement and searched databases for data published between 1999 and 2023. The scoping review aimed to answer the question: “In patients with a diagnosed and treated CGCG, able to receive an implant, does the CGCG tumor recur before or after implant surgery”? Only articles that described cases where patients with a diagnosed CGCG received an implant in a site previously affected by CGCG were included.

**Results:**

The review describes seven case reports and one case series that discuss implantology‐driven restoration after CGCG exeresis in humans. The patients, aged between 7 and 80 years, underwent surgical removal of CGCG and received implant‐supported prosthetic rehabilitation. A total of 34 implants were placed between 4 and 60 months after the tumor‐resection surgery. No recurring lesions were observed during the follow‐up period, which ranged between 2 and 12 years.

**Conclusions:**

Based on the limited evidence available, it appears that implant placement after CGCG removal is safe after a minimum of 4 months of healing. However, further research is necessary to confirm this conclusion.

## 1 Introduction

Central giant cell granuloma (CGCG) is a rare benign tumor that accounts for approximately 7% of maxillary tumors (Barthélémy and Mondié 2009; De Lange and Van den Akker 2005). Although it can occur in all maxillofacial bones and is especially observed in the maxillary bones (Suárez‐Roa et al. 2009; Whitaker and Waldron 1993), it is most commonly found in the anterior mandibular or maxillary regions (Berger et al. 2017) and predominantly affects young female patients aged 20 to 30 years (Motamedi et al. 2007).

CGCG belongs to the family of polymorph tumors, along with cherubism, brown hyperparathyroid tumors, and aneurysmal bone cysts (Barthélémy and Mondié 2009). Initially named “reparative granuloma” by Jaffe in 1953 (Barthélémy and Mondié 2009), this tumor is a reactive lesion that represents a local healing or repairing process (Dangore‐Khasbage 2015). Although the etiology of CGCG is still unknown, it may result from trauma or bone hemorrhage. The genetic cause of CGCG is currently under question due to its association with Noonan syndrome, cherubism, or type 1 neurofibromatosis (Barthélémy and Mondié 2009). According to the World Health Organization, CGCG is an intraosseous lesion that consists of cellular fibrous tissue containing multiple foci of hemorrhage and fibrosis, aggregations of multinucleated giant cells resembling osteoclasts, hemosiderin clusters, and some trabeculae of woven bone (Barthélémy and Mondié 2009).

Another nosological entity: the peripheral giant cell granuloma (PGCG) shares the histological features of CGCG, but is exophytic and can be found on the gingiva or alveolar ridge (Motamedi et al. 2007; Morais et al. 2019; Baesso et al. 2019). At all ages, local irritation is found to be mainly responsible for the occurrence of PCGC (Motamedi et al. 2007). Due to the different localization of the two granulomas, only CGCG will be studied in this scoping review.

Two forms of CGCG exist: nonaggressive and aggressive (Barthélémy and Mondié 2009; Soni, Phulari, and Shah 2022). The clinical situation, radiographic and histological features, as well as the treatment plan, differ accordingly. The nonaggressive form is often asymptomatic with a slow progression, and adjacent anatomic structures are displaced but not altered (Barthélémy and Mondié 2009; Suárez‐Roa et al. 2009; Berger et al. 2017; Motamedi et al. 2007; Dangore‐Khasbage 2015). In contrast, the aggressive form progresses rapidly and is typically associated with pain, paresthesia, root resorption, and cortical bone thinning or perforation (Barthélémy and Mondié 2009; Suárez‐Roa et al. 2009; Berger et al. 2017; Motamedi et al. 2007; Dangore‐Khasbage 2015). In both cases, nonaggressive and aggressive, the lesion requires treatment as it is not self‐repairing and will continue to grow. Malignant forms have been described in only 1.8% of cases (Kruse‐Lösler et al. 2006). Additionally, CGCG reoccurs in up to 49% of patients (Kruse‐Lösler et al. 2006).

Although there are examples of medical treatment described in the literature, including intra‐lesional administration of corticosteroids, systemic administration of calcitonin, α2A‐interferon, or even bisphosphonates and radiotherapy (Suárez‐Roa et al. 2009; Pogrel 2012, 2003), their efficiency in treating CGCG has yet to be proven, whether complemented by surgery or without surgical procedures. Therefore, surgical removal remains the gold standard for CGCG treatment. For small nonaggressive tumors, a simple curettage may be sufficient (Berger et al. 2017). However, in cases of extended aggressive lesions, large resections may be necessary (Berger et al. 2017). In both situations, lesion excision may result in bone defects and tooth loss, requiring rehabilitative treatment to restore good aesthetics and function.

Modern implantology, pre‐implant surgery, and implant‐supported prosthesis may offer solutions for complex rehabilitations (Tosco et al. 2009). However, the protocol for implant treatment after CGCG is still unclear (Tosco et al. 2009; Cossío et al. 2007d).

Implant‐driven rehabilitations are important for improving patients' quality of life after CGCG‐excision surgery, particularly in cases with significant tissue loss. However, it is important to consider whether implant surgery could be an additional risk factor for recurring CGCG lesions. There is no consensus on how to functionally reconstruct bone defects resulting from CGCG resection, to the best of the author's knowledge. Although immediate bone reconstruction may be advisable for large tumor excisions (Lee et al. 2008) to improve function and appearance. In addition, the timing of implant surgery or any other surgeries should be carefully considered and discussed. As any trauma can potentially cause CGCG or CGCG recurrence (Dangore‐Khasbage 2015; Kruse‐Lösler et al. 2006; de Lange et al. 2004) any surgical intervention must be well timed and rigorously performed The consequences of a secondary reactive lesion on an implanted site are significant in terms of tissue loss for the patient (Brown et al. 2015).

Due to the limited scientific data available on implantology rehabilitation after CGCG removal surgery, a scoping review of the literature is necessary. The aim of this scoping review is to clarify the rehabilitation protocol for CGCG sites by outlining the timing and relevance of implant surgery and prosthetic rehabilitation.

## 2 Materials and Methods

This scoping review follows the PRISMA‐ScR (Preferred Reporting Items for Systematic reviews and Meta‐analyses extension for Scoping Reviews) recommendations (Page et al. 2021; Munn et al. 2018). The research protocol was established beforehand, and the analysis method was described. Ethics approval and informed consent were not required for this scoping review.

The objective of this review was determined after adapting the PICO (patient intervention comparison and outcome) principle according to PRISMA‐ScR. The “Comparison” point was excluded as it was not relevant to this study. The defined population for this study includes patients diagnosed with CGCG who are eligible for implant surgery. Cases of PGCG have been excluded. The intervention being investigated is implant surgery in a site previously affected by CGCG. The expected outcome is the recurrence of the tumor after implant surgery. The rehabilitation protocol requires the following parameters: surgical technique, associated reconstructive surgery, medical treatment, time elapsed between CGCG removal surgery and implant surgery, time elapsed from implant surgery to prosthetic rehabilitation, number and type of implants, and loading protocol. This information was extracted from each article to develop recommendations for the rehabilitation of CGCG sites.

In summary, this scoping review searched for all relevant scientific literature on implantology‐driven restoration after CGCG exeresis in humans and included all types of communications except patents, conference reports, and literature reviews.

The search strategy involved an electronic search of the MEDLINE, Asian, Latino, and Russian databases via PubMed, Lillacs, Scielo, and Web of Science. Additionally, a manual search of the bibliographic references in all selected articles and Google Scholar research was conducted.

The databases above were searched using the keywords “implant” AND “central giant cell granuloma” OR “central giant cell granuloma CGCG” OR “central giant cell granulomas” OR “central giant cell granulomas CGCG” OR “central giant cell granulomata” OR “central giant cell lesion” OR “central giant cell lesions” OR “central giant cell reparative” OR “central giant cell reparative granuloma“ OR “central giant cell reparative granuloma of jaw” OR “central giant cell reparative granulomas”.

All communications with an English title published before July 1, 2024, have been identified. Communications with full text in languages other than French or English have been translated into French. If the full text was not available online, an email was sent to the corresponding author to have the full text. After removing duplicate items, the selection process involved three steps. Firstly, articles were screened by title, and then by reading the abstract. Eligibility was determined after reviewing the full‐text lecture.

To be included, articles had to meet the following criteria:

– the article described cases where patients with diagnosed CGCG received an implant in a site previously affected by CGCG.
– the article was written in English, French, Russian, or Spanish.

The following situations lead to article exclusion:

– the cases where PGCG or other tumors were described.
– articles describing peri‐implantitis if it was not associated with CGCG.
– articles not concerning implant‐supported rehabilitation.
– the articles were patents, conference reports, and literature reviews.

To avoid wrongful exclusion of relevant scientific articles, two of the authors, (T.R. and T.R.) have independently performed the same research strategy. In cases of disagreement, the decision of inclusion or exclusion was taken by F.B. and O.L. after discussion.

Data extraction was performed independently by the two authors, with a data table. Every debate on data interpretation was solved by F.B. and O.L. by means of discussion. If any data was missing, the corresponding author was contacted through email. If data was still missing, there was no exclusion of the article, instead, “missing data” was mentioned in the lecture table.

The following information was retrieved from the selected articles: name of the first author and year of the publication; age and gender of the patients along with outlines of medical history; chief complaint; the existence of an anatomopathological analysis (from the biopsy or after exeresis); the existence of a blood test; localization of the tumor (maxillary or mandibular) and/or tooth number; type of treatment: medical or surgical and their protocol; recurrence of the tumor before and after implant placement; interval between removal surgery and implant surgery; type and number of implants; implant loading protocol; type of prosthetic rehabilitation and the follow‐up period and recurrence.

No statistical analysis was performed due to the scarcity and discrepancy of the extracted data. The extracted data was presented in a table, and summaries of the results found were presented in written form.

## 3 Results

The search strategy identified 590 articles. After removing duplicates and screening based on title and abstract, 26 articles were assessed for eligibility through full‐text analysis.

Several articles were excluded from the study. One article was excluded as a duplicate due to a slight modification of the author's name (Cossío et al. 2007a). Two additional articles by the same author were excluded as duplicates, as they were Spanish versions of already‐included articles (Cossío et al. 2007b; Cossío et al. 2007c). Some articles were related to a different type of tumor (Brown et al. 2015; Mordini 2019; Hariri 2010; Galindo‐Moreno et al. 2016; Cloutier et al. 2007; Kramer et al.^ 2005^; Chiapasco et al. 2006), full text was unavailable for some articles (Alkan 2005; Kumar 2018; Hubácek et al. 2008), two articles were about CGCG appearing after implant surgery in a previously healthy site (Hirshberg et al. 2003; Atarbashi‐Moghadam et al. 2018), one article did not report implantation in a CGCG site (Halperin‐Sternfeld et al. 2016), and another article did not describe any prosthetic rehabilitation (Castillo‐Camacho et al. 2012).

**Figure 1:**
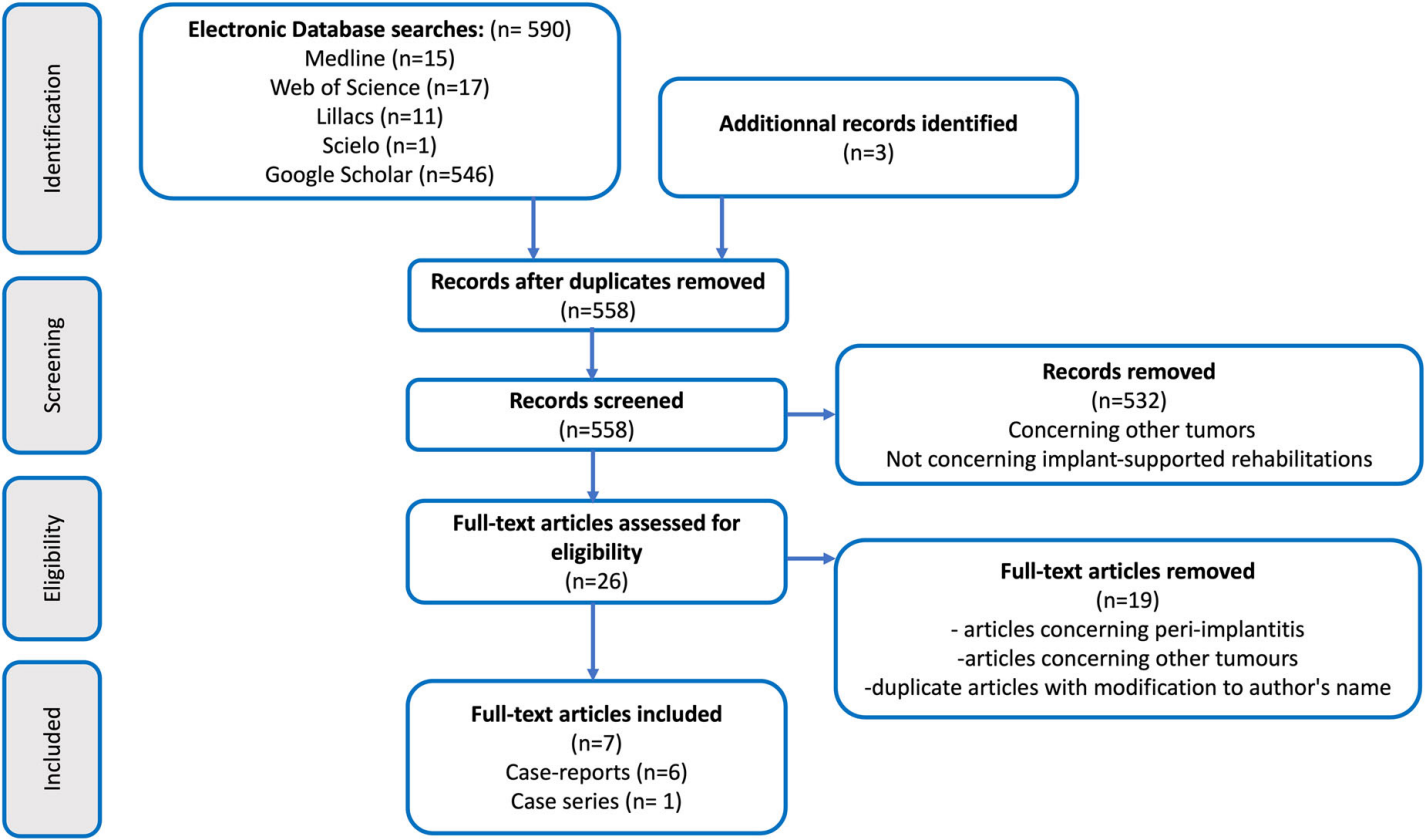
Scoping review flowchart (Page et al. 2021).

Data was extracted from seven articles, including six case reports and a case series, which treated a total of 24 patients ranging in age from 7 to 80 years, Figure 1, Table 1.

**Table 1:** Studies associated with implant rehabilitation after CGCG tumor removal.

Author, Year		Chavis et al. 2018		Yuzbasioglu et al. 2014		Lee et al. 2008		Cossio et al. 2007d		Saxena, et al. 2014		Nogueira et al. 2016		Tosco, 2009 (Tosco et al. 2009)	
Article type		Case report		Case report		Case report		Case report		Case report		Case report		Case series	
Gender, age, and medical history of the patient		Female 9 y.o.		Female 9 y.o.		Male 12 y.o.		Female 48 y.o.; tooth mobility and extraction		Male 24 y.o.; medical treatment for infection		Female 20 y.o.		3 males, 15 females, age 7 to 80 y.o. Abnormal Ca and HPT patients excluded	
Chief complaint and localization		Recurrent lesion with sinusal complications		Lower incisors mobility, buccal swelling		Pain, sublingual tumor, lower incisor mobility for 1 month		Pain and swelling of for 3 months, tooth mobility		Progressive swelling without infection signs, tooth		Important painful, rapidly growing swelling with teeth movement;		10 patients: facial and buccal swelling;4 patients: pain, tooth mobility, rapid swelling, paresthesia;	
Localization		Maxillary		Mandibula; 33 to 46 teeth		Mandibular bone, angle to angle		Mandibula, 37 tooth		Mandibula, 31 to 36		Mandibula, symphysis, and left body		12 cases: maxillary bone; 6 cases: mandibula	
Anatomo‐pathological analysis	Blood test	Yes	NO	Yes Biopsy	Yes	Yes Biopsy	NO	Yes Biopsy	Yes	Yes Biopsy	NO	Yes Biopsy	YES	Yes	YES
Diagnosis		Aggressive CGCG		CGCG		CGCG		CGCG		CGCG		Aggressive CGCG		11 aggressive and 7 nonaggressive CGCG	
Treatment Surgical/Medical		Surgical; Local exeresis, Caldwell‐Luc		Surgical; Aggressive curettage		Surgical; Mandibular resection angle to angle		Surgical, Exeresis within healthy margins		Surgical, Complete curettage with adjacent teeth extraction		Medical (corticosteroids then calcitonin) then surgical		Surgical; Bloc exeresis within healthy margins	
Grafting		Autograft		Autograft		Autograft		NO		Autograft		Reconstruction plate		Autograft	
Recurrence before implant treatment		None		None		None		Yes, after 12 months; excision and graft		None		None		None	
Delay before implant surgery		5 years		4 years		0.75 years		2 years		0.5 years		1 year		0.3 years	
Implant		Nobel active BioCare regular		Zimmer Swiss plus tapered		Straumann		Osseotite NT (3i implants)		Pitt Easy Innova Oraltronics		Biomet 3i		DM	
Number of implants		3		3		6		2				7		12	
Loading protocol		Immediate		Conventional, data missing		Conventional, 5 months		Conventional, 3 months		Conventional, 6 months		Data missing		Data missing	
Follow‐up period with no CGCG recurrence and implant survival		1 year		Data missing		9 months		5 years		2 years		12 years		2 to 10 years	

Abbreviations: DM, Data missing; Follow‐up period, no recurrence and implant survival, y.o., years old.

All patients underwent surgical removal of CGCG and received implant‐supported prosthetic rehabilitation. Only four of the patients were male. Most patients had limited medical history. The mandibular bone was the location of the lesion in 17 of the 24 patients. Although biopsy before surgery was only performed in five patients, a blood test was conducted on 21 out of 24 patients.

An anatomopathological analysis was performed in 6 cases. The diagnosed CGCG lesions were classified as either aggressive (13 cases) or nonaggressive (7 cases). In 4 cases, the aggressive character of the lesion was not reported.

Generally, surgical treatment was administered, except in one case where medical treatment with corticosteroids and calcitonin failed, and surgery was performed subsequently. Most excision surgeries resulted in an autograft due to significant tissue loss. In the two cases where autograft was not immediately performed, there was either no grafting or autografting in a second surgical time.

In total, at least 34 implants from various brands were placed at intervals ranging from 4 to 60 months after tumor‐resection surgery. The loading protocol was conventional, with no immediate or early loading reported.

During the follow‐up period, which ranged from 2 to 12 years, no recurring lesions were observed. However, in one patient, the CGCG tumor recurred 12 months after resection surgery but before implant placement.

## 4 Discussion

This scoping review analyzed seven scientific articles, including six case reports and one case series, out of a total of 586 articles. The aim was to determine the optimal timing of implant‐driven rehabilitation in patients who had suffered from CGCG tumors to avoid recurrent lesions.

Due to the lack of guidelines for CGCG, even experienced surgeons must make a judgment call regarding temporization after tumor removal. The main question raised by this review is: what is the healing time required before the next reconstruction or implant surgery after CGCG excision that leads to implant success?

The analysis of the 24 cases in the selected articles confirmed the general demographic trends associated with CGCG tumors. Although the population was generally young, CGCG tumors occurred in patients of all ages, as previously described. The data collected showed a clear preference for females, with the maxillary and mandibular bones being the most affected. However, no conclusions could be drawn regarding the etiology of the lesions, and there was no specific patient history associated with them. The main complaint was usually swelling, and sometimes pain and/or tooth mobility.

Although an anatomopathological analysis is necessary for a correct diagnosis of the tumor, blood tests were not always performed. Blood tests are crucial in the differential diagnosis of CGCG with brown hyperparathyroidism tumors due to their histopathological resemblance. If implant‐driven rehabilitation is planned, it is recommended to ensure normal levels of calcium, alkaline phosphatase, and phosphorus (Tosco et al. 2009). This is because surgical intervention and bone loss may be unnecessary in cases of hyperparathyroidism (Pogrel 2012, 2003; Hussain and Hammam 2016).

All reported cases have been treated surgically, except for one case in which medical treatment was attempted but failed. The efficacy of medical treatments, such as calcitonin, steroids, or interferon, has not yet been discussed, but successful medical treatment has been reported (Pogrel 2012, 2003). Medical treatment should be considered to avoid extensive tissue loss and large excisions. However, due to the lack of complete understanding of the treatment mechanisms, it is recommended that treatments such as calcitonin be reserved for recurring or multilocular lesions (Pogrel 2012, 2003).

The 24 cases reported here did not show any recurrent lesions after implant placement. In the single case where a recurrent lesion was present before implant placement, at 12 months (Cossío et al. 2007d), the initial excision surgery was performed using a retromolar approach and did not require any bone reconstruction. Following the second curettage surgery, an autograft was necessary. It is noteworthy that all implants were placed in a grafted site. The risk of CGCG recurrence may be affected by the placement of the implant. If the implant is placed in an autologous peroneal, iliac, or scapular graft, it would not be at risk for CGCG recurrence. This is because the lesion mostly affects facial bones.

In this scoping review, the healing time after tumor excision ranged from 4 to 60 months. However, a direct relationship between the healing time and the absence of recurrent lesions cannot be established due to insufficient data. This can be explained by the fact that the incidence of the lesion is rare in the general population (De Lange and Van den Akker 2005). As a consequence, there are few mentions of CGCG in scientific literature. Additionally, the large range of time elapsed between the removal surgery and implant placement is coherent with the patient's age. In cases where a young patient has a large, deformative lesion, it is common sense to remove it as soon as possible. Implant placement should be delayed until adulthood, or at least after the growth peak (Shah et al. 2013). Therefore, in cases such as those described by Chavis or Yuzbasioglu (Chavis et al. 2018; Yuzbasioglu et al. 2014), the bone loss occurring after CGCG removal surgery at 9 years old was fully rehabilitated after five years, in adolescence. In adults, however, a minimum of 4 months of healing was allowed (Tosco et al. 2009).

In 2004, De Lange (de Lange et al. 2004) described the recurrence rates of CGCG in the general population of the Netherlands. According to this author, patients over 20 years old had a lower recurrence rate compared to younger patients, and by the fifth year after removal surgery, a recurrence rate of up to 13.9% was expected. Therefore, it is important to consider the potential risk of a recurrent tumor when deciding on implant placement timing. Previous research indicated that implant placement should be postponed for 24 months after excision surgery (Whitaker and Waldron 1993). However, in the retrieved case reports, implant placement 4 to 6 months after CGCG removal did not result in CGCG recurrence. In addition, complete bone healing after an extraction surgery, for example, occurs from 4 weeks after the respective surgery (Udeabor et al. 2023). As CGCG tumor resection should be performed within healthy tissue, it seems safe to presume that bone healing would occur similarly. Thus, a shorter healing time in adults should not negatively impact the risk of recurrent lesions.

Although no recurrent CGCG lesions were found in this scoping review, cases of recurrent tumors with giant cells after implant placement have been described, for PGCG lesions (Brown et al. 2015; Atarbashi‐Moghadam et al. 2018). While mostly found in soft tissue, PGCG shares the histologic features of CGCG (Baesso et al. 2019). By analogy, regular check‐ups are mandatory for patients with CGCG antecedents.

This scoping review has a number of limitations in direct connection with the rare character of this benign tumor. The review has identified only case reports, which offer valuable insights but have inherent limitations. Due to their focus on individual experiences, case reports lack control groups and cannot definitively assess treatment effectiveness. Furthermore, the limited number of cases included may not capture the full spectrum of the phenomenon under investigation.

## 5 Conclusions

Within the limits of this scoping review, it can be concluded that implant placement following CGCG removal is safe after at least 4 months of healing. However, further research is required.

## Author Contributions

Study conception and design: Roman Tatiana, Robert Thomas, Leclercq Olivier, Nafash Gilbert, Boschin Francois. Data collection: Roman Tatiana, Robert Thomas, Leclercq Olivier, Kharouf Naji, Boschin Francois. Analysis and interpretation of results: Roman Tatiana, Robert Thomas, Leclercq Olivier, Nafash Gilbert, Kharouf Naji, Olivier Etienne, Boschin Francois. Draft manuscript preparation: Roman Tatiana, Robert Thomas, Leclercq Olivier, Nafash Gilbert, Kharouf Naji, Olivier Etienne, Boschin Francois. All authors reviewed the results and approved the final version of the manuscript.

## Ethics Statement

The authors have nothing to report.

## Consent

The authors have nothing to report.

## Conflicts of Interest

The authors declare no conflicts of interest.

## Data Availability

The data that support the findings of this study are available from the corresponding author upon reasonable request.
